# Social media for dissemination and public engagement in neurosurgery—the example of Brainbook

**DOI:** 10.1007/s00701-018-3757-8

**Published:** 2018-12-10

**Authors:** A. Alamri, P. Rogers, C. Kearns, T. Doke, A. Al-Habib, F. Servadei, P. J. Hutchinson, A. G. Kolias, Chris Uff

**Affiliations:** 1 Department of Neurosurgery, The Royal London Hospital, London, UK; 2 Royal Infirmary of Edinburgh, United Kingdom and Artibiotics, University of Edinburgh, Edinburgh, UK; 3 Faculty of Medicine, King Saud University, Riyadh, Saudi Arabia; 4 Department of Neurosurgery, Humanitas University and Research Hospital, Milan, Italy; 5 World Federation of Neurosurgical Societies, Nyon, Switzerland; 6 NIHR Global Health Research Group on Neurotrauma, University of Cambridge, Cambridge, UK; 7 Department of Clinical Neurosciences, University of Cambridge & Addenbrooke’s Hospital, Cambridge, UK; 8 Queen Mary University of London, London, UK

**Keywords:** Brainbook, Neurosurgery, Global surgery, Public engagement, Science dissemination

## Abstract

**Background:**

Public engagement has become one of the most effective tools in gaining feedback and perspectives from members of the public, involving patients with decisions, and inspiring young people to carry the medical profession forwards. Brainbook is a multi-platform, social media-based resource that was created specifically to enhance public engagement in neurosurgery and results from one of its case discussions will be reported in this paper.

**Methods:**

A Brainbook case was created in collaboration with the NIHR Global Health Research Group on Neurotrauma and presented over 3 days (23–25 February 2018). YouTube videos were created depicting the management of an acute subdural haematoma using patient interviews, medical illustration, consultant-led discussion and operative footage. Content was shared across all Brainbook social media platforms and analytics were gathered through social media applications.

**Results:**

Over a 72-hour time period, and across multiple social media accounts, 101,418 impressions were achieved (defined as penetrance onto individual media feeds and total views of the content), with active discussion on social media.

**Conclusions:**

Neurosurgical content published across multiple social media outlets represents an encouraging and exciting potential for global engagement across multiple audiences. Social media can be an effective method of not only disseminating neurosurgical knowledge, but activating and engaging the public, allied healthcare professionals, medical students and neurosurgeons.

**Electronic supplementary material:**

The online version of this article (10.1007/s00701-018-3757-8) contains supplementary material, which is available to authorized users.

## Introduction

Public engagement is an umbrella term that encompasses and promotes the sharing of ideas, opinions, practice and activities between communities and specialists. Active participation, debate and discussion from both parties generate meaningful dialogue giving specialists a better understanding of public opinion and perspective, whilst also introducing the public to the current practices and advances in different specialist fields [7, 8]. This is of particular relevance in medicine and surgery where the vocation is built on serving the public. More recently, clinical decision making has evolved through the concept of ‘shared decision making’, moving the profession away from paternalism and towards a more patient-centred approach [3]. The UK MAGIC (Making Good Decisions In Collaboration) programme identified that patient activation and public engagement is one of the key methods of embedding shared decision making into standard patient care [6]. The study recognised that health outcomes were much improved when patients were actively involved, and that health resources were utilised more effectively and efficiently [6].

Public engagement in medicine and surgery has become one of the most effective tools in gaining feedback and perspectives from members of the public, involving patients with decisions, and inspiring young people to carry the profession forwards. It may also serve to improve relationships between specialists and the public, to improve the doctor-patient rapport, and to increase trust from patients, friends and family members, and other members of the public [1, 4, 13]. Engaging the public equips and empowers them to take control of their health, and to harness the opportunity to change health attitudes and behaviours [5]. The implications of public engagement in neurosurgery are especially pertinent. It is a specialty of which little is known or understood by non-specialists. Emergency work and trauma accounts for greater than 50% of the neurosurgical workload and due to the urgency of these procedures, there is little time to fully explain the pathology, the procedure, and likely outcomes to patients and their families [2]. In these emergency situations, every attempt is made to explain and discuss the pathology and treatment options in the limited time available, yet most decisions will still be made by neurosurgeons in the patient’s best interests, with discussion taking place retrospectively. In a specialty where outcomes can be devastating, it is of primary concern that every attempt is made to promote discussion and sharing of perspectives outside of the operating theatre and the emergency department. Brainbook is the first neurosurgical platform dedicated to public engagement. It is an online multimodal neurosurgical resource that utilises social media to discuss cases and provides insight into life at the United Kingdom’s (UK) busiest neurosurgical major trauma centre, the Royal London Hospital, with particular highlights of the activities of the multidisciplinary team. Brainbook places emphasis on using lay terms and providing definitions for terminology to allow everyone to participate in discussions. Professional medical illustrators are employed to create engagement material relating to specific neurosurgical pathologies. Social media conversations are pitched at levels appropriate for everyone from members of the public to neurosurgeons around the world.

## Methods

The initiative encourages patients who have previously undergone a neurosurgical procedure to share their experiences, as well as their friends and relatives. The Brainbook team has collaborated with a medical illustrator, Dr. Ciléin Kearns (CK) (Artibiotics), to provide high-quality medical art and animation, in order to illustrate concepts that may be difficult to grasp.

A Brainbook case was created in collaboration with the NIHR Global Health Research Group on Neurotrauma and presented over 3 days (23–25 February 2018). YouTube videos were created depicting the management of an acute subdural haematoma using patient interviews, medical illustration, consultant-led discussion and operative footage. A lay summary of the Rescue ASDH trial was also created as depicted in the supplementary material (online resource 1). Content was shared across all Brainbook social media platforms and analytics were gathered through the individual social media applications.


A film created to explain the purpose of the Rescue ASDH trial. Commentary provided by a Consultant Neurosurgeon. (MOV 254 mb)


## Results

**Fig. 1:**
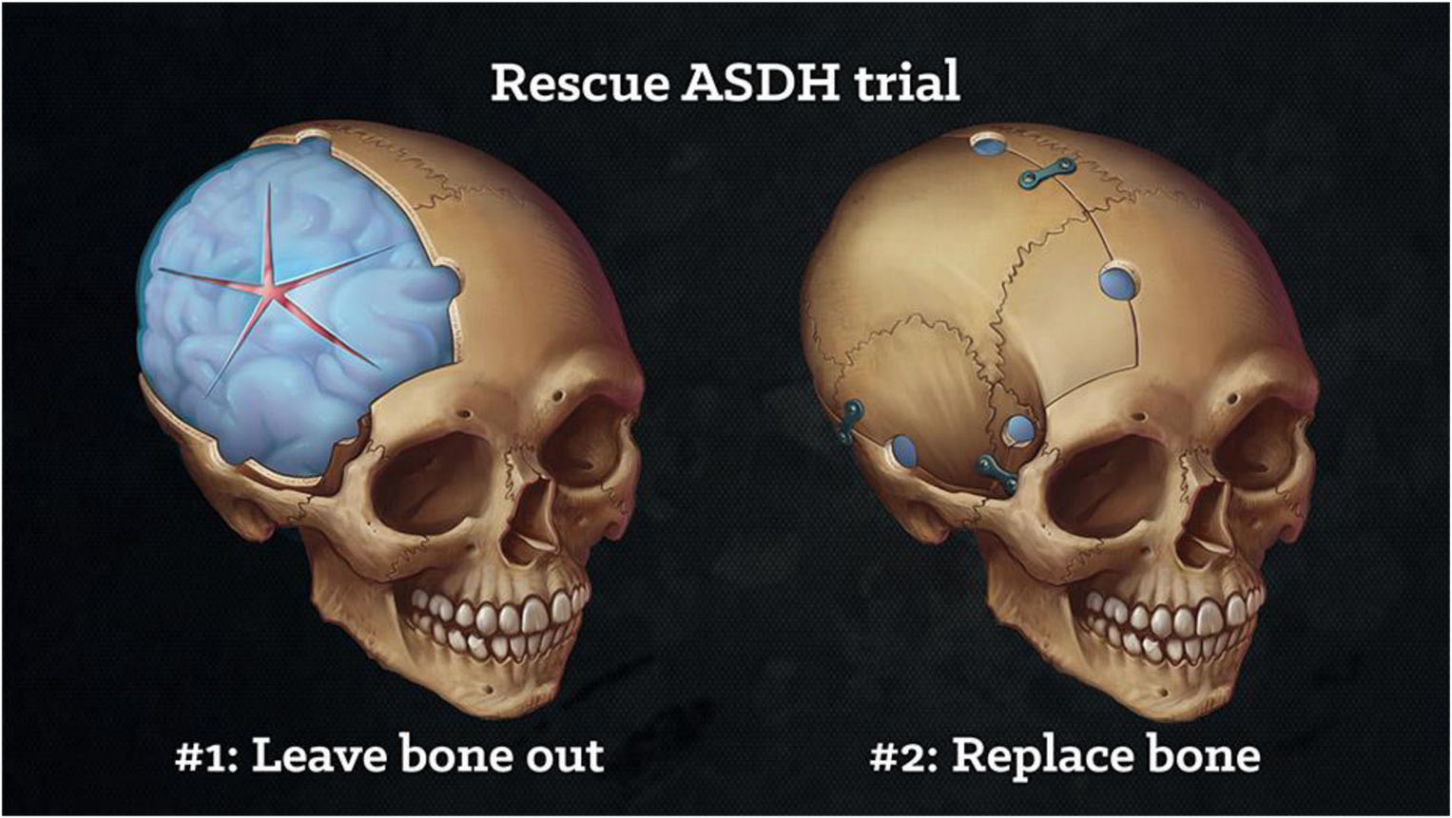
Medical illustration created to depict craniotomy versus decompressive craniectomy in the Rescue ASDH trial

The analytics showed diverse and widespread engagement, with several thousand impressions (defined as penetrance onto individual media feeds and total views of the content) through Twitter, YouTube and Instagram applications over the course of the 3 days (see Fig. 1). The most popular tweets included medical illustrations and YouTube videos, and the majority of users were below the age of 34 years.

**Fig. 2:**
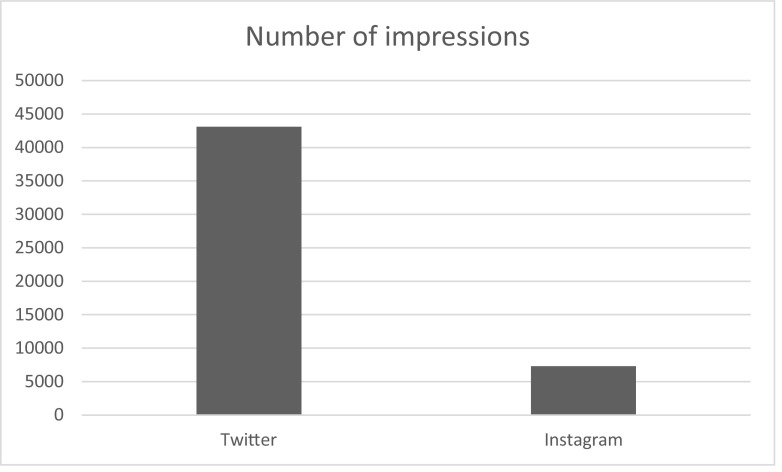
Number of impressions on Twitter and Instagram over the 3 days during which the case was ‘live’. These were generated by 106 tweets on Twitter, and 4 sets of illustrations published on Instagram

Twitter was utilised as the main social media channel for discussion of the case. On Twitter, a total of 106 tweets generated 43,100 impressions (Fig. 2). The most popular tweets included links to medical illustrations on Instagram and YouTube videos. Fifty-four percent of the Twitter users were aged 13–17 years old (range 13–65) and the male/female ratio was 54:46.

The main YouTube video had 3293 views over 72 h with 61% of viewers originating in the United States (US). This was the most popular Brainbook video on YouTube (5320 min watched over 72 h).

Four sets of medical illustrations were published on Instagram over the 72 h, which generated 7290 impressions. Forty-seven percent of users were aged 25–34 (range 13–65+). The male/female ratio was 52:48, with users predominantly in the US (21%), United Kingdom (UK) (19%) and Brazil (10%).

The images were also published on the social media accounts of the collaborating medical illustrator (CK) in the same time frame. Six Instagram posts generated 25,482 impressions in users’ Instagram feeds and the posts were discovered by 18,886 new users. Six companion posts on Twitter reached 17,420 users. On Reddit, seven posts achieved 7412 impressions, with a link to the YouTube video being the most popular medical post for a period of 24 h. In total, there was a reach of 51,028 through the Artibiotics combined social media platforms. Brainbook and Artibiotics combined impressions were 101,418 over 72 h.

## Discussion

**Fig. 3:**
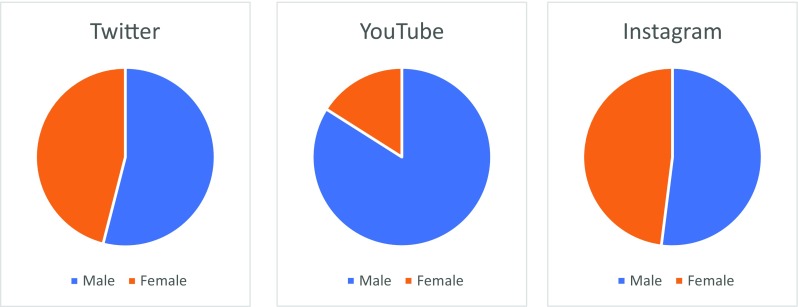
Gender of Brainbook users

The ASDH neurotrauma case was very well received, with content being viewed by over 100,000 people in just 72 h, highlighting that visual elements play an effective role in engaging a non-specialist audience, and that social media is effective in disseminating science globally. From our experience, the male-to-female ratio for the more visual elements of the cases, in particular the YouTube videos, is disproportionately favoured by male viewers, yet the illustrations and discussions are more equally attended (Fig. 3). This may reflect assumptions held by the general public and can be targeted with future Brainbook cases.

The data that was gathered was over a 72-h time period. This does not reflect the ultimate potential of the generated content. The material will continuously accrue “views” over time, especially as more content is added; however, audience growth only continues to progress as long as relevant, high-quality content is continuously created and published across social media. This represents a significant challenge, as filming and medical illustration requires significant funding and time allocation.

Work still needs to be done in addressing barriers to participation in public engagement initiatives by health professionals, and to assess why certain groups of the general public may perceive neurosurgery according to traditional stereotypes. There are a plethora of dissemination platforms that could be used, including mobile applications (apps). A good example of this is the UpSurgeOn apps which use three-dimensional (3D) renderings of anatomy as well as virtual reality (VR) and augmented reality (AR) to teach medical professionals neurosurgery. Together with Brainbook, these apps have most recently been used to disseminate information from the World Federation of Neurosurgical Societies (WFNS) in order to reach a global audience.

In sharing ideas and opinions in this way, public engagement initiatives in neurosurgery may begin to address preconceived barriers or stereotypes by informing an increasingly diverse group of people including patients, their families, the wider public, students and other health professionals [10]. These efforts may also encourage a wider selection of people to enter the medical profession or the specialty and help to improve inter-departmental relationships in a hospital setting [12]. These public engagement initiatives may also work to widen the global neurosurgical footprint, further expose the specialty and build new bridges for future science collaboration [9, 11]. Most importantly, these public engagement initiatives can allow neurosurgeons to understand the patient perspective, assist them with identifying patient concerns and help to empathise with them. Building trust and improving rapport will inevitably lead to better understanding and acceptance, and improved health outcomes. This dialogue also informs effective design of tailored resources for patients, and training for practitioners.

Perhaps in the near future, public engagement will feature within the realm of clinical and research governance, thus highlighting its importance for both clinical and academic advancements in the healthcare sector.

## Conclusions

This collaborative case between Brainbook and the NIHR Global Health Research Group on Neurotrauma, published across multiple social media outlets, represents an encouraging and exciting potential for global engagement across multiple audiences. The project has shown that social media can be an effective method of activating and engaging the public, allied healthcare professionals, medical students and neurosurgeons. This is an important opportunity to develop practices and improve the care we provide by learning from others, made more achievable on a global scale by the use of the internet and social media.
